# Maternal Vitamin D Deficiency During Pregnancy Alters Hepatic Metabolism in Adult Female Offspring Without Overt Metabolic Dysfunction

**DOI:** 10.3390/metabo16070503

**Published:** 2026-07-17

**Authors:** Miyu Isogai, Norihiro Imai, Tadashi Ogawa, Yumi Hayashi

**Affiliations:** 1 Department of Integrated Health Sciences, Nagoya University Graduate School of Medicine, Tokai National Higher Education and Research System, Nagoya 461-8673, Japan; isogai.miyu.w6@s.mail.nagoya-u.ac.jp (M.I.); norihiro.imai@gmail.com (N.I.); 2 Department of Legal Medicine, Aichi Medical University School of Medicine, Nagakute 480-1195, Japan; ogawatd@aichi-med-u.ac.jp

**Keywords:** vitamin D deficiency, developmental origins of health and disease (DOHaD), maternal, pregnancy, offspring, metabolomics, metabolic disorders, liver, mouse

## Abstract

**Background/Objectives**: Vitamin D deficiency (VDD) is a major global health concern. Although maternal VDD during pregnancy may influence metabolic health in offspring, previous studies have focused predominantly on male offspring, leaving its effects in females insufficiently characterized. This study aimed to comprehensively assess hepatic metabolic profiles in female offspring exposed to maternal VDD during gestation. **Methods**: Pregnant 129/Sv mice were fed either a control diet or a vitamin D-deficient diet throughout pregnancy. After weaning, female offspring were maintained on a normal diet and analyzed at 12 weeks of age following a 16 h fast. Hepatic metabolomic profiling was conducted using GC–MS/MS, followed by multivariate analysis. To evaluate the potential contribution of fasting, publicly available liver RNA-seq data from ad libitum-fed and 16 h-fasted mice (GSE130127) were also analyzed. **Results**: No overt metabolic abnormalities were detected between the groups. However, principal component analysis revealed differences in hepatic metabolic profiles between the Control and VDD groups. Levels of 4-aminobutyric acid and proline were significantly elevated in the VDD group. Pathway analysis revealed significant alterations in arginine and proline metabolism and purine metabolism, along with changes in pathways associated with amino acid and energy metabolism. Comparison with fasting-associated liver RNA-seq data revealed minimal overlap, although the potential influence of fasting cannot be completely excluded. **Conclusions**: Maternal VDD during pregnancy was associated with altered hepatic metabolic profiles in female offspring despite the absence of overt metabolic abnormalities. These findings suggest that maternal VDD may influence hepatic metabolism in female offspring and indicate that the potential long-term effects of maternal VDD on offspring metabolism warrant further investigation.

## 1. Introduction

Vitamin D plays an essential role in numerous physiological functions beyond the maintenance of bone health. In humans, it is synthesized in the skin following exposure to ultraviolet B radiation from sunlight or acquired through dietary intake [1,2,3]. By binding to nuclear receptors expressed in a wide range of tissues, vitamin D regulates gene expression throughout the body [4]. Nevertheless, vitamin D deficiency (VDD) remains highly prevalent worldwide because of inadequate dietary intake and limited exposure to sunlight [3,5]. VDD has traditionally been recognized for its contribution to skeletal disorders, including rickets, osteomalacia, and osteoporosis. More recently, growing evidence has linked VDD to an increased risk of chronic diseases, including cardiovascular disease, type 2 diabetes (T2D), and metabolic disorders [6,7]. During pregnancy, vitamin D is transferred across the placenta to the fetus; consequently, maternal VDD leads to fetal VDD [3,8]. According to the Developmental Origins of Health and Disease (DOHaD) concept, prenatal VDD may also increase susceptibility to adverse health outcomes later in life [9].

The DOHaD concept was originally proposed by David J. P. Barker, who demonstrated an association between adverse fetal conditions and an increased risk of cardiovascular disease in adulthood [10,11]. Since then, numerous studies have explored the long-term health consequences of prenatal environmental exposures within the DOHaD framework, including maternal VDD. Several reports have suggested that maternal VDD during pregnancy may adversely affect metabolic health in adult offspring, most commonly resulting in increased fat mass, insulin resistance, hypertension, and altered lipid metabolism [12,13,14,15]. However, many previous studies either did not evaluate male and female offspring separately or focused exclusively on males [15,16]. Although some investigations have assessed sex-specific effects, several have reported that female offspring appear less susceptible, potentially because of the protective effects of estrogen [17]. At the same time, the traditional view that female mice should be excluded from experimental studies owing to variability associated with the estrous cycle has increasingly been questioned [18,19]. Furthermore, the conclusion that female offspring are protected from the effects of prenatal VDD is often based on a limited set of representative outcomes. Consequently, potential effects on other, unexamined biological parameters cannot be ruled out.

To address this gap, we focused on hepatic metabolism because the liver plays a central role in the regulation of glucose, lipid, and amino acid metabolism and is a key organ involved in maintaining metabolic homeostasis [20]. Previous studies have suggested that maternal VDD may influence metabolic health in offspring; however, its effects on hepatic metabolic profiles, particularly in female offspring, remain poorly understood [12,13,14,15,16,17]. Therefore, we performed a comprehensive metabolomic analysis of the liver to determine whether maternal VDD during pregnancy alters hepatic metabolic profiles in adult female offspring.

## 2. Materials and Methods

### 2.1. Animal Procedures

All animal experiments performed in this study were conducted following the guidelines of the Nagoya University Committee on Animal Use and Care (Approval No. D240040-002, D250027-002).

**Figure 1:**
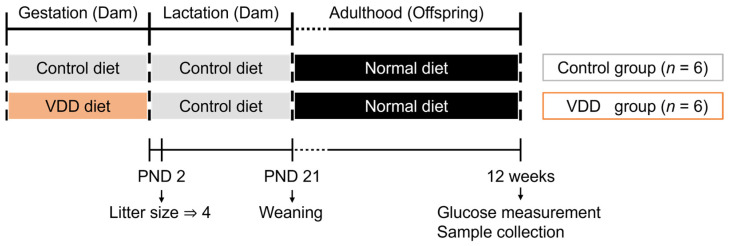
Schematic overview of the animal experimental design. During gestation, pregnant dams were assigned to either a control diet or a vitamin D-deficient diet. During lactation, all dams were fed a control diet. Litter sizes were standardized to four pups per litter on postnatal day (PND) 2. Following weaning, offspring were maintained on a normal diet. Fasting blood glucose measurements and sample collection were performed at 12 weeks of age (*n* = 6 per group). The dotted line indicates a break in the timeline between PND21 and 12 weeks of age.

An overview of the animal experimental design is presented in Figure 1. All 129/Sv mice used in this study were generated by in-house breeding and housed under controlled environmental conditions (25 °C, 12 h light/dark cycle) without direct exposure to natural sunlight, with free access to chow and water. Healthy female and male mice aged 10 weeks were paired for mating. For timed mating, females were examined daily for the presence of a vaginal plug, and pregnancy was confirmed upon plug detection. During gestation, pregnant dams were randomly allocated to one of two dietary groups: a control group fed a control diet (AIN-93G, vitamin D3 = 1000 IU/kg) or a vitamin D-deficient group fed a vitamin D-deficient diet, which was custom-prepared based on the AIN-93G formulation and was identical to the control diet in nutrient composition, including caloric content, calcium, and phosphorus, except for vitamin D3 content (vitamin D3 = 25 IU/kg; CLEA Japan Inc., Tokyo, Japan). During lactation, all dams received the control diet.

At birth, all offspring remained with their biological dams until weaning, and litter sizes were standardized to four pups per litter on postnatal day (PND) 2. To confirm vitamin D status, serum 25(OH)D3 concentrations were measured in offspring at PND2 using the Cayman Vitamin D ELISA Kit (Item No. 501050; Cayman Chemical, Ann Arbor, MI, USA). Because of the limited serum volume available at this age, serum samples were pooled prior to analysis. Offspring were weaned at PND21, at which time body weight was recorded. All subsequent analyses were conducted exclusively in female offspring. A total of six female offspring per group were included in the analyses. Female offspring were selected from five litters in the Control group and four litters in the VDD group. To minimize litter effects, offspring were selected from multiple litters, and no more than two female offspring from a single litter were included. After weaning, offspring were maintained on a normal diet (CE2, CLEA Japan Inc., Tokyo, Japan) *ad libitum* throughout adulthood, and food intake was monitored weekly. At 12 weeks of age, offspring underwent a 16 h fast, after which body weight was measured and fasting blood glucose concentrations were determined using a LaboGluco device (Research & Innovation Japan Inc., Chiba, Japan). Subsequently, the animals were euthanized, and liver tissues and serum samples were collected. The liver was excised and weighed. For histological analysis, a portion of the liver was fixed in 10% neutral-buffered formaldehyde, whereas the remaining liver tissue and serum samples were snap-frozen and stored at −80 °C until further analysis.

### 2.2. Histological Analysis

Following fixation in formalin, liver tissues were dehydrated through a graded ethanol series and infiltrated with paraffin. The tissues were subsequently embedded in paraffin and sectioned at a thickness of 3 μm using a sliding microtome (Leica, Wetzlar, Germany). Sections were stained with hematoxylin and eosin according to standard protocols and examined using a BZ-9000 microscope (Keyence, Osaka, Japan). Images were acquired using the same system.

### 2.3. Biochemical Tests

Serum biochemical parameters, including aspartate aminotransferase (AST), alanine aminotransferase (ALT), triglycerides (TG), phospholipids (PL), free fatty acids (FFA), total cholesterol (T-Cho), esterified cholesterol (E-Cho), and free cholesterol (F-Cho), were analyzed by SRL Inc. (Tokyo, Japan).

### 2.4. Sample Preparation and GC–MS/MS Analysis for Metabolomic Profiling

Sample preparation and GC–MS/MS analysis were performed according to a previously published protocol with minor modifications [21]. Liver tissues (82 ± 9 mg; Control, *n* = 6; VDD, *n* = 6) were homogenized in 1.2 mL of a methanol:water:chloroform solvent mixture (2.5:1:1, *v*/*v*) containing 72 µL of 0.5 mg/mL 2-isopropylmalic acid as an internal standard. After shaking at 37 °C and centrifugation, the aqueous phase was collected, mixed with distilled water, and centrifuged again. Finally, the resulting supernatant was concentrated using a centrifugal vaporizer before being freeze-dried overnight.

Methoximation was carried out using methoxyamine hydrochloride in pyridine at 30 °C for 90 min, followed by trimethylsilylation with N-methyl-N-trimethylsilyltrifluoroacetamide at 37 °C for 30 min. In addition, we also prepared 10-fold diluted samples to ensure reliable detection of highly abundant metabolites. Next, GC–MS/MS analysis was performed using a GCMS-TQ8040 gas chromatograph–tandem mass spectrometer (Shimadzu, Kyoto, Japan). Samples were automatically injected in split mode using a split ratio of 1:30 for undiluted samples and 1:300 for 10-fold diluted samples. The order of GC–MS/MS measurements was randomized using a random number generator to minimize potential batch effects.

### 2.5. Data Processing and Multivariate Analysis

Metabolites were identified by matching retention times and two multiple reaction monitoring transitions using GCMSsolution software (version 4.51, Shimadzu, Kyoto, Japan). Peak areas were normalized to the internal standard (2-isopropylmalic acid) and tissue weight prior to multivariate analysis. All multivariate analyses, including principal component analysis (PCA), partial least squares discriminant analysis (PLS-DA), volcano plot generation, and pathway analysis, were performed using MetaboAnalyst 6.0 and R version 4.4.0.

Specifically, PCA was conducted according to a standard procedure, and was used to visualize relationships among samples [22]. Before analysis, data were log10-transformed to normalize their distribution. Auto-scaling (mean-centering and division by the standard deviation of each variable) and PCA were performed using the *prcomp* function as implemented in R. To visualize differences in data distribution between groups, 95% confidence ellipses were superimposed on PCA plots using the ellipse function.

Next, PLS-DA was performed to identify metabolites contributing to group discrimination. Following normalization using the same procedure applied for PCA, auto-scaling and PLS-DA were conducted using the *mixOmics* package. PLS-DA score plots and variable importance in projection (VIP) score plots were generated with *ggplot2*. VIP score plots were used to identify metabolites contributing to group discrimination. Metabolites with VIP scores ≥ 1.0 were considered important contributors to group discrimination. To evaluate the stability of the PLS-DA model, cross-validation was performed using the *perf* function in the *mixOmics* package with five-fold cross-validation repeated 100 times. Model performance was assessed based on classification error rates and balanced error rates (BER).

Volcano plots were generated using *ggplot2* to visualize metabolites that were significantly altered between groups. Fold changes (FC) were calculated as the ratio of the mean metabolite abundance in the VDD group to that in the Control group and are presented as log2-transformed values (log2FC). For metabolite comparisons, *p*-values were calculated using Welch’s two-tailed *t*-test. To account for multiple testing, *p*-values were adjusted using the Benjamini–Hochberg procedure, and the resulting false discovery rate (FDR) values were used for significance assessment. Metabolites with |log2(FC)| > 1 and FDR < 0.1 were considered significantly altered.

Pathway analysis was performed in MetaboAnalyst 6.0 using all identified metabolites. Specifically, pathway enrichment analysis was conducted using the Global Test. In addition, pathway topology analysis was performed by comparing the relative betweenness centrality values based on the *Mus musculus* KEGG pathway library.

### 2.6. Quantitative Real-Time PCR

Total RNA was extracted from liver tissue using the PureLink^®^ RNA Mini Kit (Thermo Fisher Scientific K.K., Tokyo, Japan). Complementary DNA (cDNA) was synthesized and quantitative real-time PCR (qPCR) was performed using the GoTaq^®^ 2-Step RT-qPCR System (Promega K.K., Tokyo, Japan) according to the manufacturer’s instructions. Amplification was carried out using a QuantStudio™ 1 Real-Time PCR System (Thermo Fisher Scientific K.K., Tokyo, Japan) under the following cycling conditions: 95 °C for 2 min, followed by 40 cycles of 95 °C for 3 s and 60 °C for 30 s. A melting curve analysis was performed to confirm the specificity of amplification. Relative mRNA expression levels were calculated using the 2^−ΔΔCt method with *Gapdh* as the internal control. The primer sequences (5′→3′) were as follows: *Abat*, Forward–CTGAACACAATCCAGAATGCAGA Reverse–GGTTGTAACCTATGGGCACAG; *Prodh*, Forward–CAGCCTCATTGACAGCAG Reverse–CTCCTCAGTGAACCGTGAC; *Pycr2*, Forward–CTCCTGTATCCGAACCAGA Reverse–GGGTGACTCCAGCTTCACT; *Gapdh*, Forward–AGAACATCATCCCTGCATCCA Reverse–CCGTTCAGCTCTGGGATGAC [23,24].

### 2.7. RNA-Seq

To compare the metabolomic alterations observed in this study with fasting-associated transcriptional responses, publicly available mouse liver RNA-seq data were obtained from the GEO database under accession number GSE130127 [25]. Liver RNA-seq datasets from ad libitum-fed (AL) and intermittent fasting 16 h (IF16) mice (*n* = 6 per group) were analyzed. Sequence Read Archive files were downloaded from the corresponding dataset, and paired-end FASTQ files were generated using fasterq-dump with the split-files option (https://github.com/ncbi/sra-tools, accessed on 1 May 2026). Raw FASTQ files were subsequently analyzed using the BioJupies platform (https://maayanlab.cloud/biojupies/, accessed on 6 May 2026). Data processing, differential expression analysis, and pathway enrichment analysis were performed using the default BioJupies workflow and parameters. KEGG pathway enrichment analysis was conducted using the Enrichr-based pathway enrichment module implemented in BioJupies.

### 2.8. Statistical Analyses

Group comparisons of body weight, food intake, liver weight, fasting blood glucose concentrations, individual metabolite levels, and relative mRNA expression levels were performed using Welch’s unpaired two-tailed *t*-test. Statistical analyses of metabolite levels were conducted using the original values, whereas graphical representations were generated using values normalized to the mean of the Control group and expressed as relative abundance. All data are presented as mean ± standard error of the mean (SEM). All statistical analyses were performed using R version 4.4.0, and bar graphs were generated with GraphPad Prism version 9.5.1 (GraphPad Software, San Diego, CA, USA). Statistical significance was defined using a two-sided threshold of *p* < 0.05.

## 3. Results

### 3.1. Physiological Characteristics of Offspring

Serum 25(OH)D3 concentrations measured at PND2 were lower in the VDD group than in the Control group (Figure S1). Because serum samples were pooled prior to analysis, only a limited number of independent samples were available for analysis, and statistical analysis was not performed.

**Figure 2:**
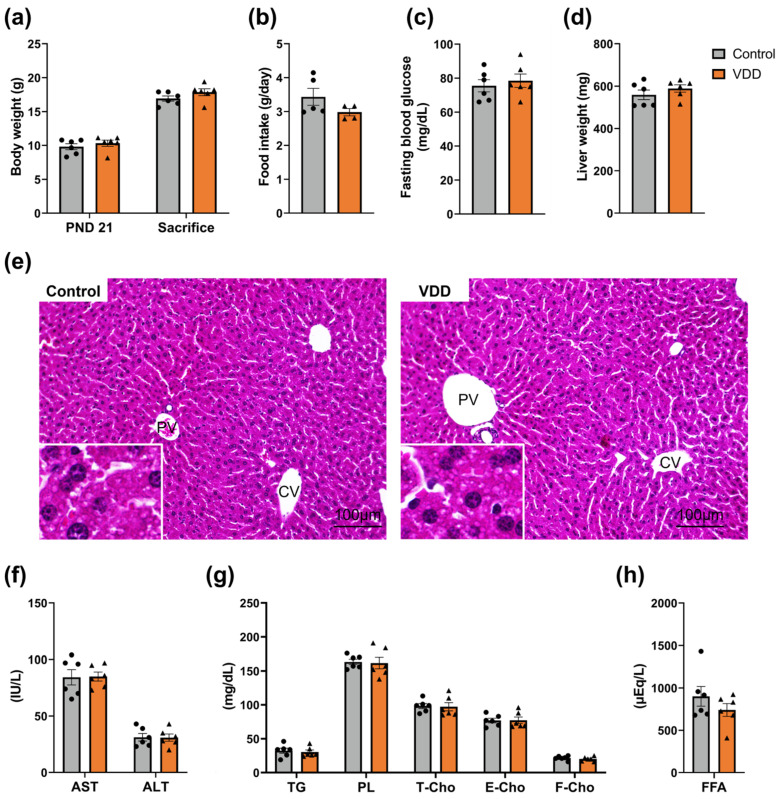
General phenotypic characterization of Control and VDD offspring. (**a**) Body weight of mice at postnatal day 21 and before sacrifice in each group (**g**). No significant differences were observed at either PND21 or before sacrifice. (**b**) Average food intake from weaning to sacrifice (g/day). (**c**) Fasting blood glucose concentrations (mg/dL). (**d**) Liver weight (mg). (**e**) Representative hematoxylin and eosin (H&E)-stained liver sections (scale bar = 100 μm). Enlarged views are shown in the lower left corner. PV, portal vein; CV, central vein. (**f**) Serum biochemical parameters, including aspartate aminotransferase (AST) and alanine aminotransferase (ALT). (**g**) Serum biochemical parameters, including triglycerides (TG), phospholipids (PL), total cholesterol (T-Cho), esterified cholesterol (E-Cho), and free cholesterol (F-Cho). (**h**) Serum free fatty acid (FFA) concentrations. Data are presented as mean ± SEM, and each dot represents an individual sample (●, Control; ▲, VDD).

There were no significant differences in body weight between the Control and VDD groups at either PND21 (Control: 9.8 ± 0.44 g vs. VDD: 10.33 ± 0.45 g, *p* = 0.424) or the time of sacrifice (Control: 16.92 ± 0.38 g vs. VDD: 17.85 ± 0.51 g, *p* = 0.176) (Figure 2a). Similarly, no significant differences were observed in food intake (Control: 3.43 ± 0.25 g/day vs. VDD: 2.98 ± 0.11 g/day, *p* = 0.152), fasting blood glucose concentrations (Control: 75.5 ± 3.62 mg/dL vs. VDD: 78.5 ± 3.96 mg/dL, *p* = 0.588), or liver weight (Control: 558.67 ± 22.89 mg vs. VDD: 588.83 ± 17.3 mg, *p* = 0.320) between the groups (Figure 2b–d).

Histological examination of liver sections revealed no signs of inflammation in either group. Microvesicular steatosis due to fasting was observed in the hepatic lobules of both groups; however, no apparent histological differences were observed between the Control and VDD groups (Figure 2e), in which the portal vein (PV) and central vein (CV) are indicated.

Serum biochemical analyses revealed no significant differences in AST, ALT, TG, PL, T-Cho, E-Cho, F-Cho, or FFA levels between the Control and VDD groups (Figure 2f–h).

### 3.2. PCA and PLS-DA

**Figure 3:**
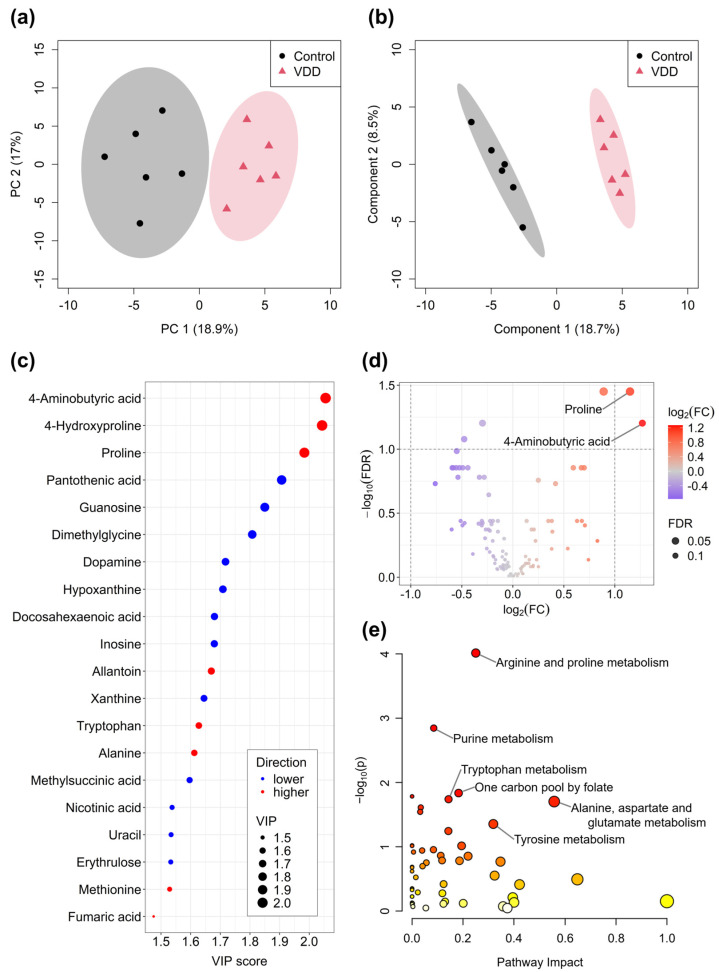
Liver metabolomic profiles of Control and VDD offspring. (**a**) Principal component analysis (PCA) score plot based on metabolite profiles showing the first two principal components (PC1 and PC2). Each point represents an individual sample, and colors and shapes indicate the experimental groups. Ellipses represent the 95% confidence intervals for each group. (**b**) Partial least squares discriminant analysis (PLS-DA) score plot based on metabolite profiles showing the first two components (Components 1 and 2). Each point represents an individual sample, and colors and shapes indicate the experimental groups. Ellipses represent the 95% confidence intervals for each group. (**c**) Top 20 metabolites ranked by variable importance in projection (VIP) scores derived from PLS-DA. Each dot represents a metabolite. Dot size corresponds to the VIP score, with larger dots indicating higher VIP scores. Dot color indicates the direction of change in the VDD group relative to the Control group (red, higher in VDD; blue, lower in VDD). (**d**) Volcano plot showing differentially abundant metabolites between the Control and VDD groups. The *x*-axis represents log2(fold change [FC]), and the *y*-axis represents −log10(false discovery rate [FDR]). (**e**) Pathway analysis scatter plot of liver metabolites. The *x*-axis represents pathway impact, and the *y*-axis represents −log10(p). Dot size reflects pathway impact, whereas dot color indicates the level of statistical significance.

The untargeted metabolomics analysis identified a total of 113 compounds with known biochemical identities (Table S1). As shown in Figure 3a, PCA revealed a tendency toward separation between the Control and VDD groups along the first principal component (PC1). To further explore metabolic differences between the Control and VDD groups, PLS-DA was performed and showed a tendency toward separation of samples from the Control and VDD groups (Figure 3b). To identify metabolites contributing to this discrimination, VIP scores were calculated. A total of 45 metabolites exhibited VIP scores ≥ 1.0, a commonly used threshold for important contributors. The top 20 metabolites ranked by VIP score are presented in Figure 3c and included 4-aminobutyric acid, 4-hydroxyproline, amino acids (e.g., proline, tryptophan, alanine, and methionine), purine-related metabolites (e.g., guanosine, hypoxanthine, inosine, allantoin, and xanthine), and organic acids such as fumaric acid. To assess model stability, five-fold cross-validation repeated 100 times was performed. The resulting BER were 0.043 and 0.039 for the first and second components, respectively.

### 3.3. Volcano Plot

Changes in metabolite levels and their statistical significance between the Control and VDD groups were evaluated based on FC and FDR and visualized using a volcano plot (Figure 3d). Two metabolites, 4-aminobutyric acid and proline, exhibited significant differences between the Control and VDD groups, and both metabolites were significantly elevated in the VDD group.

### 3.4. Pathway Analysis

Pathway analysis identified several altered metabolic pathways, with arginine and proline metabolism showing the strongest enrichment (*p* = 0.0000965, FDR = 0.00492), followed by purine metabolism (*p* = 0.00142, FDR = 0.0362) (Figure 3e). Additional pathways, including one carbon pool by folate, tryptophan metabolism, alanine, aspartate, and glutamate metabolism, and tyrosine metabolism, also reached nominal significance (*p* < 0.05); however, these pathways did not remain significant after correction for multiple testing. Among the identified pathways, alanine, aspartate, and glutamate metabolism exhibited the highest pathway impact value, whereas arginine and proline metabolism showed the strongest statistical enrichment.

**Figure 4:**
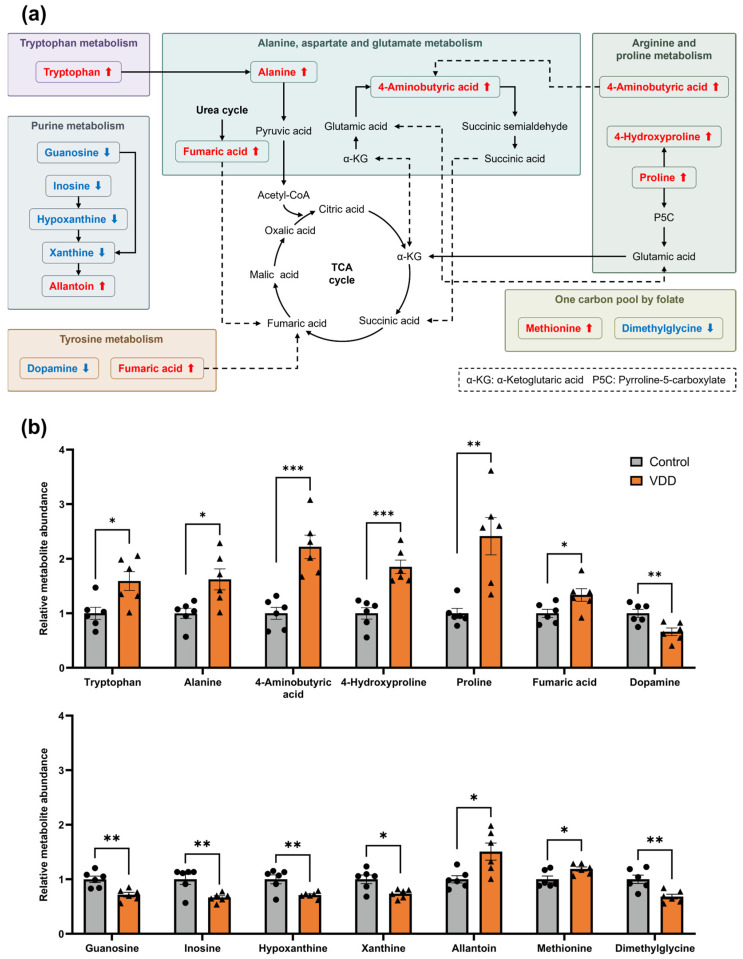
Diagram showing altered metabolic pathways and associated metabolites in the liver of VDD offspring. (**a**) Schematic overview of the altered metabolic pathways and associated metabolites. Metabolite labels and arrows indicate the direction of change in the VDD group relative to the Control group (i.e., red/upward arrows, higher in VDD; blue/downward arrows, lower in VDD). Black solid arrows indicate the direction of metabolic pathways, and black dashed lines connect metabolites shared between different pathways. (**b**) Relative abundances of metabolites associated with the altered metabolic pathways. Data are presented as mean ± SEM, and each dot represents an individual sample (●, Control; ▲, VDD). * *p* < 0.05, ** *p* < 0.01, *** *p* < 0.001 (unadjusted *p*-value).

To further characterize the pathways identified in the pathway analysis, individual metabolites associated with the enriched pathways were examined (Figure 4a,b). Within arginine and proline metabolism, levels of 4-aminobutyric acid, 4-hydroxyproline, and proline were increased in the VDD group. In contrast, purine metabolism was characterized by reduced levels of guanosine, inosine, hypoxanthine, and xanthine, together with increased levels of allantoin. Within alanine, aspartate, and glutamate metabolism, alanine, 4-aminobutyric acid, and fumaric acid were elevated in the VDD group. Several metabolites, including 4-aminobutyric acid and fumaric acid, were shared among multiple pathways.

### 3.5. qPCR

To further investigate genes involved in the arginine/proline metabolism pathway, the mRNA expression levels of *Abat*, *Prodh*, and *Pycr2* were analyzed by qPCR. No significant differences were observed between the Control and VDD groups (Figure S2).

### 3.6. RNA-Seq

We first analyzed publicly available liver RNA-seq data from AL and IF16 mice to compare fasting-associated transcriptional responses with the metabolomic alterations observed in offspring exposed to maternal VDD. KEGG pathway analysis identified 11 significantly altered pathways after FDR correction and 64 nominally significant pathways based on raw *p*-values (*p* < 0.05) (Table S2). Among the pathways identified in the metabolomic analysis, only one carbon pool by folate overlapped with the nominally significant pathways detected in the RNA-seq analysis, whereas no overlapping pathways remained after FDR correction.

## 4. Discussion

In this study, we conducted a comprehensive metabolomic analysis of the liver to investigate the effects of maternal VDD during pregnancy on adult female offspring. Previous studies have focused predominantly on male offspring, whereas relatively few have examined females [12,15,16]. To our knowledge, this is the first study to comprehensively characterize hepatic metabolites in female offspring within this context. Although several studies have suggested that female offspring may be protected against metabolic alterations because of the effects of estrogen [17], our findings indicate that maternal VDD may also influence metabolic profiles in females. These results provide new insight into the effects of maternal VDD on adult female offspring.

In the present study, no significant differences were detected between the Control and VDD groups in body weight, food intake, fasting blood glucose concentrations, liver weight, liver histology, or serum biochemical parameters, including lipid profiles and liver enzymes. These findings are consistent with previous reports and suggest that maternal VDD did not induce overt metabolic abnormalities in female offspring. Nevertheless, metabolomic analyses suggested differences in hepatic metabolic profiles between the Control and VDD groups.

PCA revealed a tendency toward separation between the Control and VDD groups along PC1. PCA is an unsupervised multivariate approach that identifies orthogonal components accounting for the greatest variance within a dataset [26]. Because PCA does not incorporate group labels, the observed separation reflects intrinsic differences in the underlying data structure among samples. Accordingly, this finding suggests that maternal VDD exposure alters metabolic profiles in female offspring.

To further evaluate group discrimination using a supervised approach, PLS-DA, which incorporates group labels and maximizes separation between groups [27], was performed, and VIP scores were calculated. VIP analysis identified 45 metabolites with VIP scores > 1, including several amino acid- and purine-related metabolites, suggesting that these compounds may contribute to the metabolic alterations associated with maternal VDD exposure. However, multivariate analyses do not directly assess the statistical significance of individual metabolites. Therefore, statistical analyses based on FC and FDR were performed, and the results were visualized using a volcano plot to identify significantly altered metabolites between groups. Two metabolites, 4-aminobutyric acid and proline, were significantly elevated in the VDD group. 4-Aminobutyric acid is widely recognized as an inhibitory neurotransmitter that is predominantly distributed in the mammalian central nervous system. However, 4-aminobutyric acid is also present in peripheral tissues, including the liver, although at lower levels [28]. Previous studies have shown that 4-aminobutyric acid administration exerts beneficial effects, including hepatoprotection [29], suppression of obesity [30], and improvement of glucose metabolism [31]. In contrast, increased endogenous hepatic 4-aminobutyric acid production has been associated with insulin resistance, T2D, and increased body mass index (BMI) [23]. Proline, on the other hand, is a glucogenic amino acid that enters glutamate metabolism and can serve as a substrate for gluconeogenesis. An experimental study in mice demonstrated that proline reduces paraspeckle formation in hepatocytes, thereby promoting gluconeogenesis and contributing to hyperglycemia [32]. Collectively, these findings suggest that the elevated levels of 4-aminobutyric acid and proline observed in the VDD group may reflect alterations in amino acid metabolism and glucose homeostasis. To further investigate the metabolic pathways associated with these metabolite changes, pathway analysis was performed.

Pathway analysis revealed alterations in several metabolic pathways, including arginine and proline metabolism, alanine, aspartate, and glutamate metabolism, and purine metabolism. These pathways are functionally linked through amino acid utilization and energy metabolism. Among the amino acid-related pathways, alanine (e.g., alanine, aspartate, and glutamate metabolism), tryptophan (tryptophan metabolism), and proline (e.g., arginine and proline metabolism) were significantly increased in the VDD group. All three are classified as glucogenic amino acids. Previous studies have reported that elevated circulating levels of several amino acids, including alanine, tryptophan, and proline, are positively associated with the future development of T2D in normoglycemic individuals, suggesting that systemic alterations in amino acid profiles may occur before the onset of overt T2D [33,34]. Therefore, the altered metabolite profile observed in the VDD group may indicate differences in hepatic metabolic regulation. However, no significant differences were observed in the mRNA expression of the selected genes involved in the arginine and proline metabolism pathway. The mechanisms underlying these metabolomic alterations and their biological significance remain to be clarified. In purine metabolism, guanosine, inosine, hypoxanthine, and xanthine levels were decreased, whereas allantoin levels were increased in the VDD group. In mice, allantoin is a downstream product of purine degradation and has been reported to reflect enhanced purine catabolism and oxidative stress. Previous studies have suggested that oxidative stress contributes to metabolic dysregulation, including insulin resistance and T2D [35]. Accordingly, the alterations in purine metabolism observed in the VDD group may reflect changes in purine turnover and related metabolic processes. Although no differences were observed in fasting blood glucose concentrations or other conventional metabolic parameters, the metabolomic alterations identified in the present study indicate differences in hepatic metabolic profiles between groups. Because direct assessments of insulin sensitivity, such as insulin measurements, glucose tolerance tests, or HOMA-IR, were not performed, the relationship between these metabolomic alterations and insulin sensitivity remains unclear, and the physiological significance of these changes requires further investigation. Notably, pathway analysis did not identify marked alterations in lipid metabolism-related pathways, and no obvious changes were detected in either histological or serum biochemical analyses. Together, these findings suggest that maternal VDD may preferentially affect amino acid metabolism rather than lipid metabolism in the liver of female offspring under the present experimental conditions.

Finally, to assess the potential contribution of fasting to the observed metabolic alterations, publicly available liver RNA-seq data from AL and IF16 mice were analyzed. In the present study, mice were sacrificed after a 16 h fast to evaluate hepatic metabolic alterations under a metabolically challenged state characterized by enhanced lipid utilization and altered glucose metabolism [20,36]. Therefore, it was possible that the metabolic changes observed in the present study were not specific consequences of maternal VDD but instead reflected an exaggerated response to fasting. Among the pathways identified in the metabolomic analysis, only one carbon pool by folate overlapped with the nominally significant pathways identified in the RNA-seq dataset, and no overlapping pathways remained after FDR correction. Although this comparison cannot exclude the possibility that fasting contributed to the observed metabolic alterations, the limited overlap between the two datasets suggests that fasting alone may not fully account for all of the metabolic changes observed in the VDD group. These findings raise the possibility that maternal VDD may influence hepatic amino acid and energy metabolism beyond fasting-related responses. However, because the RNA-seq dataset was derived from an independent study and no ad libitum-fed control group was included in the present study, these interpretations should be considered exploratory and require further investigation.

This study has several limitations. First, because this study was based on untargeted metabolomic profiling, causal relationships cannot be established and the underlying mechanisms remain unclear, although the mRNA expression of selected genes involved in the arginine and proline metabolism pathway was evaluated. In addition, the analytical approach may not comprehensively capture all metabolites because of limitations in analytical coverage and detection sensitivity. Second, the sample size was relatively small (*n* = 6 per group), and because one or two female offspring were selected from each litter, statistical power may have been limited and potential litter effects cannot be excluded. In addition, vitamin D status was assessed using a limited number of pooled serum samples from offspring at PND2, and additional measurements will be required to further validate vitamin D status. Finally, this study focused exclusively on female offspring. Therefore, the present findings should not be interpreted as female-specific effects, and additional studies including male offspring are required to determine whether the observed metabolic alterations are sex-dependent, occur at different developmental stages, or are influenced by alternative fasting conditions.

## 5. Conclusions

Maternal VDD during pregnancy was associated with alterations in hepatic metabolic profiles in adult female offspring, as revealed by comprehensive metabolomic analysis. Notably, these alterations were detected despite the absence of overt metabolic dysfunction, as assessed by fasting blood glucose concentrations, histological evaluation, and serum biochemical parameters. Alterations were observed in several amino acid- and energy metabolism–related pathways, indicating differences in hepatic metabolic profiles between groups. These findings suggest that the potential long-term effects of maternal VDD on offspring metabolism warrant further investigation, although the physiological significance of the observed metabolomic alterations remains unclear.

## Data Availability

The data presented in this study are available upon request from the corresponding author for a clear reason.

## Supplementary Materials

The following supporting information can be downloaded at https://www.mdpi.com/article/10.3390/metabo16070503/s1, Table S1: Relative abundances and statistical analysis results of all detected liver metabolites in Control and VDD offspring; Table S2: Significantly enriched KEGG pathways identified from publicly available liver RNA-seq data of AL and IF16 mice; Figure S1: Serum 25(OH)D3 concentrations in PND2 offspring from the Control and VDD groups; Figure S2: Relative mRNA expression levels of *Abat*, *Prodh*, and *Pycr2* in the livers of adult female offspring from the Control and VDD groups.
